# Construction and Validation of an Automatic Segmentation Method for Respiratory Sound Time Labels

**DOI:** 10.1111/crj.70124

**Published:** 2025-10-21

**Authors:** Jian Fan, Haoran Ni, Xiulan Chen, Yulin Duan, Wanmin Wang, Fan Xu, Yan Shang

**Affiliations:** ^1^ Department of General Practice, The First Affiliated Hospital of Naval Medical University Changhai Hospital Shanghai China; ^2^ Department of General Practice, Shanghai 411 Hospital China RongTong Medical Healthcare Group Co., Ltd. Shanghai China; ^3^ Department of Respiratory and Critical Care, The First Hospital Affiliated of Naval Medical University Changhai Hospital Shanghai China; ^4^ Daning Road Community Health Service Center Shanghai China; ^5^ Department of Evidence Based Medicine and Social Medicine, School of Public Health Chengdu Medical College Chengdu Sichuan China; ^6^ Sichuan Provincial Key Laboratory of Philosophy and Social Sciences for Intelligent Medical Care and Elderly Health Management Chengdu Medical College Chengdu Sichuan China

**Keywords:** artificial intelligence, automatic segmentation method, digital auscultation, respiratory sound, respiratory system diseases

## Abstract

**Background:**

In the field of respiratory system diseases, the utilization of respiratory sounds in auscultation plays a crucial role in the specific disease diagnosis. However, during the process of auscultation, the personal experiences and environmental factors may affect the decision making, leading to diagnostic errors. Therefore, to accurately and effectively obtain and analyze respiratory sounds can be positively contribute to the diagnosis and treatment of respiratory system diseases.

**Objectives:**

Our aim was to develop an analytical method for the visualization and digitization of respiratory audio data, and to validate its capability to differentiate between various background diseases.

**Methods:**

This study collected the respiratory sounds of patients admitted to the Department of General Medicine of Shanghai Changhai Hospital from June to December 2023. After strict screening according to the inclusion and exclusion criteria, a total of 84 patients were included. The research process includes using an electronic stethoscope to collect lung sounds from patients in a quiet environment. The patients expose their chests and lie flat. Sound data are collected at six landmark positions on the chest. The collected audio files are imported into an analysis tool for segmentation and feature extraction. Specific analysis methods include distinguishing heart sounds and respiratory sounds, segmenting respiratory sounds, determining the inspiratory and expiratory phases, and using a tool developed by the team for automatic segmentation encoding.

**Results:**

We standardized the respiratory sounds of 84 patients and segmented multiple respiratory cycles. Following the localization and segmentation of the respiratory cycles based on label information, we calculated the average and standard deviation of the amplitude features for each segment of the respiratory cycle. The results indicated differences among various diseases.

**Conclusions:**

The robust algorithm platform is capable of segmenting the respiratory sounds into inhale and exhale phases accordingly, then comparing the difference between different background diseases. This method provides objective evidence for the auscultation of respiratory sounds and visual display of breath sounds.

## Graphical abstract

A digital visualization method segments respiratory sounds into inhale/exhale phases, enabling robust analysis and comparison across diseases. It provides objective auscultation evidence and visual breath sound displays.

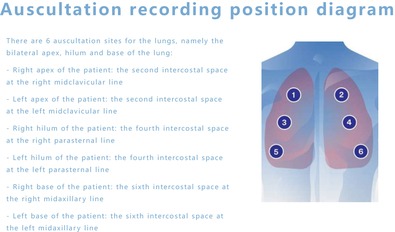

## 1 Introduction

The physiological respiratory cycle can be divided into the inhalation and exhalation phases. Inhalation causes the expansion of the lungs, allowing air to be drawn in. While the exhalation causes the contraction of the lungs, leading to the expulsion of air. The respiratory sound signal can reflect the valuable information about the physiological or pathological status of the respiratory system [1]. For instance, the abnormalities of the expiratory phase are frequently observed in conditions such as asthma, chronic obstructive pulmonary disease (COPD), lung infections, bronchiectasis, and interstitial lung disease. Moreover, the abnormalities in the inspiratory phase commonly manifest in upper respiratory tract obstruction, muscle weakness, pleural effusion, and pneumothorax. Currently, lung auscultation is widely recognized as the most robust and reliable approach for detecting pulmonary abnormalities. For example, Xu et al. proposed an innovative approach to accurately assess lung function by analyzing cough sounds recorded via mobile devices [2]. This method overcomes limitations of time and location, enabling frequent sampling and evaluation of lung function in the home environment using smartphones [2]. Furthermore, Raj et al. investigated the profound potential of utilizing lung auscultation, specifically vesicular and bronchial breath sounds, for diagnosing and addressing COVID‐19 concerns. These findings hold significant implications in the realm of medical research [3].

However, the auscultation of the lungs highly depends on the proficient respiratory specialists, and the technique relies on the acoustic manifestations of the respiratory cycle (i.e., contraction and relaxation of the respiratory muscles) to detect abnormalities in lung function. These important respiratory sounds, encompassing both the inspiratory and expiratory phases, are localized at six distinct auscultation sites on the chest surface. However, they are highly susceptible to interference throughout the respiratory cycle, potentially leading to missed detection of vital respiratory signals. Consequently, there is an urgent imperative to explore enhanced methodologies for analyzing respiratory sounds.

The rapid development of artificial intelligence enables the identification of previously undetectable patterns in medical imaging and signal data, thereby improving the diagnosis of lung diseases [4, 5, 6]. Several algorithms have been validated for the robust automation capabilities in the segmentation of respiratory sounds. For example, Huang et al. proposed a technique for the detection and classification of respiratory sounds using vesicular sounds and crackle peak detection [7]. Through quantitative analysis of respiratory sounds, clinicians can differentiate between different types of moist rales, determine their timing within the breathing cycle, and assess their severity. The study conducted by Huang et al. employed an artificial intelligence in terms of deep learning methodology to transform lung sounds into two‐dimensional spectrograms and used convolutional neural networks (CNN) for end‐to‐end recognition of respiratory system diseases or abnormal lung sounds [8]. To achieve segmentation results that align with the sequential nature of respiratory sounds, the author proposes a Python Package called TSSEARCH, which integrates subsequence search and time series similarity measurement with sequence timing models [9].

The collected respiratory sounds can be objectively analyzed through computerized intelligent analysis, allowing the signals to be decomposed into different intensities and frequencies for a more accurate characterization of such signals. This technology enables a more objective analysis of respiratory sounds and noise to better understand them [10]. Techniques used in other applications include the determination of breath sound boundaries, feature extraction of breath sounds and noise, differentiation between expiratory and inspiratory phases, and reduction of noise interference. To characterize breath sound parameters in a more accurate way, artificial intelligence algorithms will provide a more intelligent approach used in the overall deciphering process.

Complex network analysis can be applied to the study of bioacoustics signals and bronchial breath sounds. By utilizing machine learning techniques that extract graph features, the intensity and frequency of respiratory sounds can be measured, which represent fundamental characteristics of sound during the breathing process [11]. In addition, the electronic stethoscope was used to digitize respiratory sounds with full patient cooperation in a quiet environment, while providing hardware and software tools to analyze the raw data.

The present study introduced a novel approach for the automated segmentation of respiratory sounds and extraction of fundamental frequency and intensity parameters. This methodology combines the Audio Data Analysis Tool with customized Python language. The proposed method represents a significant advancement in the field of artificial intelligence analysis of respiratory sounds, making valuable contributions to the diagnosis and prediction of respiratory system diseases.

## 2 Methods

### 2.1 General Information

A total of 100 patients were initially assessed for eligibility. After strict screening based on the inclusion and exclusion criteria, 16 patients were excluded. Consequently, 84 patients were ultimately included in this study. Collect respiratory sounds of 84 patients admitted to the Department of General Medicine at Shanghai Changhai Hospital from June 2023 to December 2023.

### 2.2 Inclusion and Exclusion Criteria

Inclusion Criteria: 18 years of age or older; agree to the consent for the collection of respiratory sounds. Exclusion criteria included: unwillingness to cooperate with respiratory sound collection during the research period; the presence of congenital airway abnormalities, pulmonary underdevelopment, or other related illnesses; severe substantial organ damage preventing cooperation; currently receiving respiratory machine treatment; the presence of contagious respiratory diseases.

### 2.3 Research Process

**FIGURE 1:**
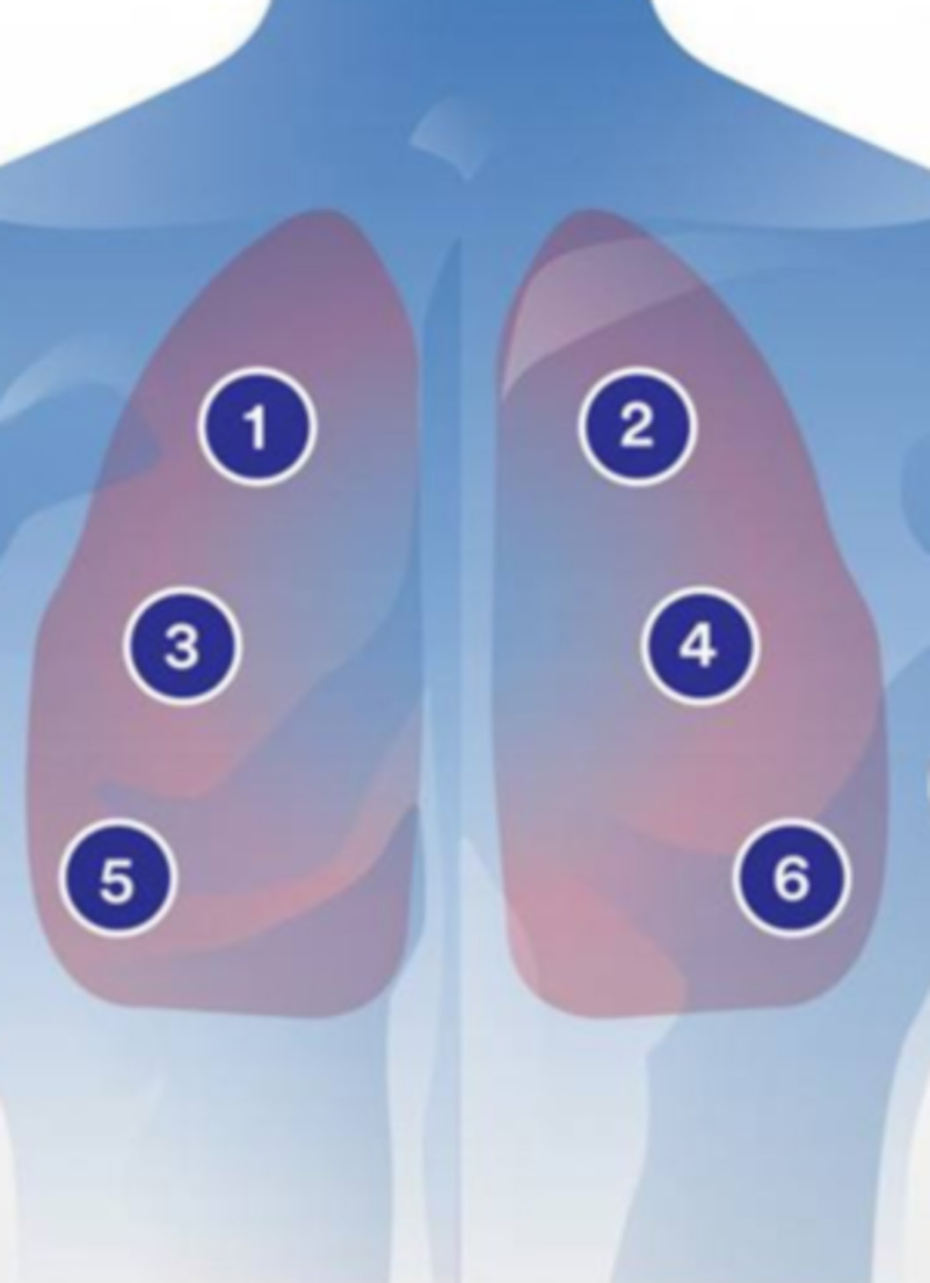
Schematic diagram of chest auscultation.

Firstly, the ETZ‐1A(C) electronic stethoscope was used to collect lung sounds from patients while maintaining a quiet environment. The patient was invited to take a supine position and expose the chest, and the electronic stethoscope was disinfected and warmed up. The stethoscope auscultation head was placed upon the recording location on the chest skin with appropriate pressure, and the respiratory sound data were acquired by using the related mobile application. The duration of each recording should cover at least two respiratory cycles in each lung auscultation zone. The auscultation of lung sounds was recorded at six different locations. These locations are as follows: right upper chest (intersection between the second rib and the midclavicular line on the right side), left upper chest (intersection between the second rib and the midclavicular line on the left side), right middle chest (intersection between the fourth rib and the anterior axillary line on the right side), left middle chest (intersection between the fourth rib and the anterior axillary line on the left side), right lower chest (intersection between the sixth rib and the anterior axillary line on the right side), and left lower chest (intersection between the sixth rib and the anterior axillary line on the left side) (Figure 1). It is important to avoid auscultating over areas where heart sounds are heard. Data from each participant were collected continuously for 3 days, with two sessions per day (8:00–11:00 and 14:30–17:00). The best audio data from one of the 3 days were selected for analysis. In our recording process, the WAV format was used (16 kHz/44.1 kHz sampling rate, 16‐bit depth, dual‐channel) for export. Among the commonly used formats for breath sound data, WAV is a lossless format that retains complete details, making it suitable for accurate analysis.

**FIGURE 2:**
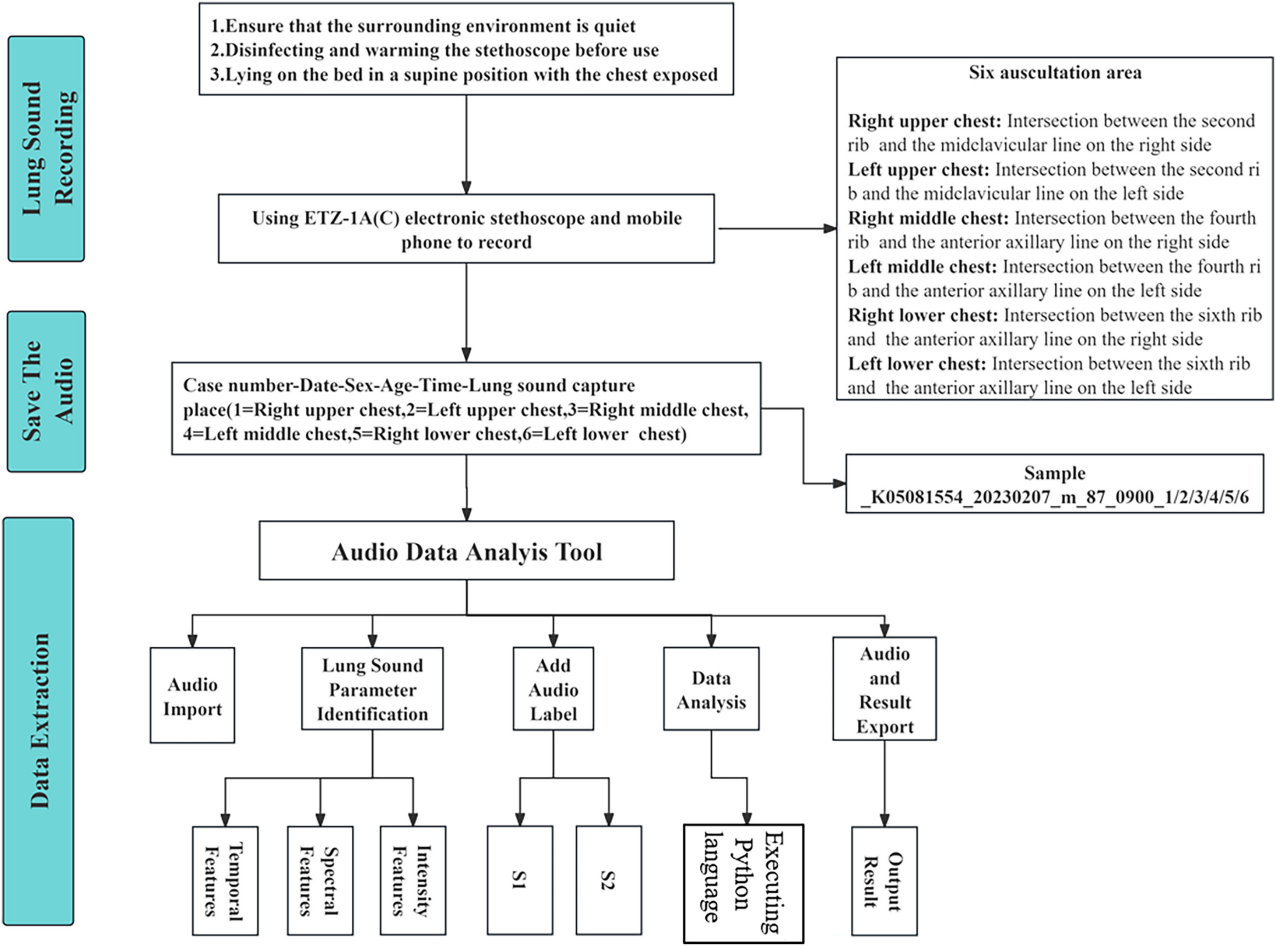
General technical workflow.

Finally, the audio files were imported into the audio data analysis tool and automatically segmented the audio data representing one cycle into two segments (inspiratory phase S1 and expiratory phase S2). Then, the detailed feature parameters were extracted from each frame of the lung sound signal, including time‐domain features (such as energy, mean value, and peak value), frequency‐domain features (such as spectral energy and frequency peak), and time‐frequency‐domain features (such as wavelet coefficients) (Figure 2).

### 2.4 The Differentiation of Cardiac and Respiratory Sounds

In the beginning, in order to remove the background noise from the heartbeat signal, uniform sampling was applied during the normalization process. Then the Short‐Time Fourier Transform (STFT) [11] was used to convert it into a frequency spectrum representation. The STFT amplitude spectrum was processed by a Recursive Neural Network (RNN) model [12] to obtain time‐frequency masks for the heart sound signal and noise signal. The RNN Noise Reduction Module is a three‐layer bidirectional LSTM architecture that was specifically designed to address two types of noises commonly found in breathing sounds: environmental noise, such as conversational sounds and equipment clutter, and physiological disturbances, such as the superimposition of heart tones. The training data were manually labeled with “noise‐clean” breath sound pairs, which include normal breath sounds and pathological sounds such as rhonchi and rales.

The STFT magnitude spectrum was multiplied by the mask output derived from the RNN, followed by signal reconstruction using inverse STFT to obtain lung sounds contaminated with noise. Subsequently, the heart sound signal was filtered and subjected to Hilbert transform for feature extraction. The heart rate and duration were calculated, and a Hidden Markov Model (HMM) was employed to obtain different sub‐state sequences of the heart sound signal. The HMM classification decision involves the implementation of a six‐state HMM model, in which the states correspond to the following respiratory cycle: inspiration onset—inspiration—inspiration end—expiration onset—expiration—expiration end. The initialization of the transfer probability matrix is based on 1000 normal respiratory cycle statistics. The HMM was initialized using priors derived from a separate dataset of 1000 normal respiratory cycles collected from healthy volunteers, which provides a strong prior on the physiological sequence of respiratory phases. This model was subsequently adapted to individual patient data using Bayesian inference.

### 2.5 The Segmentation of Respiratory Sounds

The Audio Data Analysis Tool software was utilized to visualize the respiratory sounds, ensuring that the starting point in the audio file aligns with either the inhalation or exhalation phase of breath sounds. This approach effectively eliminates the interference from breathing and external noise. The specific characteristics of sound were taken into consideration, and feature parameters were extracted from each frame of lung sound signals. These parameters include temporal features (duration), spectral features (spectral energy, frequency peak, and spectral slope), and intensity features (maximum amplitude, minimum amplitude, skewness, and kurtosis). These results were examined to identify the most consistent and distinguishable respiratory sound signals for segmentation.

### 2.6 The Segments of the Inspiratory and Expiratory Phases

A breathing cycle consists of an inhalation phase and an exhalation phase, where the inhalation phase is defined as the time from the start of S1 to the beginning of S2. The exhalation phase is defined as the time from the beginning of S2 to the start of the next breathing cycle at S1; see Table 1. The relevant audio clips were being trimmed, and the audio file was segmented into distinct sections. The customized app was constructed based on Python language to segment the audio clips and analyze the raw data.

**TABLE 1:** The detail parameters extracted.

Duration	Intensity	Frequency
S1 start time	Max dB	Start_f0
S1 end time	Min dB	Max_f0
S2 start time	Mean dB	Min_f0
S2 end time	Middle dB	Mean_f0
Respiratory cycle duration	Respiratory cycle duration dB	Middle_f0

### 2.7 The Segmentation of Inspiratory and Expiratory Phases

The amplitude and time axis of the S1 peak were automatically determined by our audio analysis data tool, based on the baseline at the end of preprocessing. Additionally, it calculated the peak value of S1 and identified the frequency range preceding the valley of the previous wave. The start and end times of S1 can be determined in this manner. Subsequently, the second peak occurring between two adjacent S1 peaks was utilized as a distinctive feature for extracting the S2 peak. The S2 peaks that significantly deviated from the average value were filtered out, followed by selecting the data with the highest statistical significance. The corresponding time of this selected data serves as a label to obtain the starting point and ending point of S2. The starting times of S1 and S2 were subsequently calculated. Finally, utilizing statistical data, the start and end times, states, decibels, amplitudes, and other relevant information for each state were computed. See Table 2.

**TABLE 2:** Calculate relevant parameters and provide descriptions.

Parameter	Description
Startf0	Starting frequency
Maxf0	Maximum‐frequency
Minf0	Minimum frequency
Meanf0	Mean frequency
Middlef0	Median frequency
Rangef0	Frequency full distance
SME	Reduction to slope from maximum to end
SSM	Slope start to max
Endf0	The frequency of the end
Jitter	Signal shaking
Harmonicity	Harmonization, harmonization
SNR	Signal noise ratio

### 2.8 Automatic Segmentation Encoding

The Respiratory Data Analysis Tool was developed based on the Python language for comprehensive audio analysis and feature extraction. It also facilitates automated generation of spectrograms and provides visual representations, preprocessed audio samples, and detailed data results.

## 3 Results

### 3.1 Population Dataset

A total of 84 patients were recruited, including 43 male individuals with an average age of (65.50 ± 9.45) years and 41 female individuals with an average age of (64.90 ± 6.48) years. The details of the demographics of the subjects refer to Table 3.

**TABLE 3:** Demographics of subjects.

Characteristic	Male (mean ± STD)	Female (mean ± STD)
Number	43	41
Heart rate	71.60 ± 4.20	68.80 ± 6.40
Blood oxygen	96.38 ± 1.23	98.00 ± 0.82
Age	65.50 ± 9.45	64.90 ± 6.48
Height	176.12 ± 5.32	159.78 ± 4.10
Weight	69.32 ± 9.86	50.78 ± 6.12
BMI	22.38 ± 3.26	19.92 ± 2.50

### 3.2 Visualization and Denoise the Respiratory Sound

**FIGURE 3:**
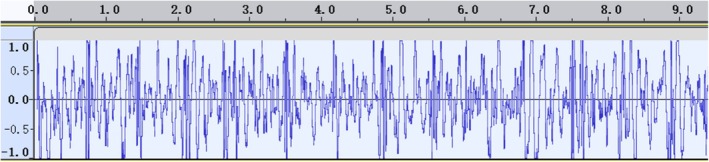
Lung sounds before denoising process (Corresponding Power: 0 dB = 1 pW).

**FIGURE 4:**
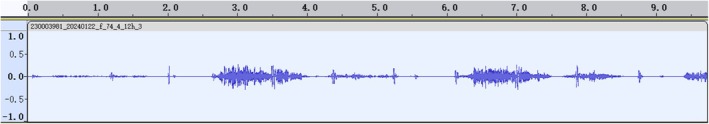
Lung sounds after denoising process (Corresponding Power: 0 dB = 1 pW).

**FIGURE 5:**
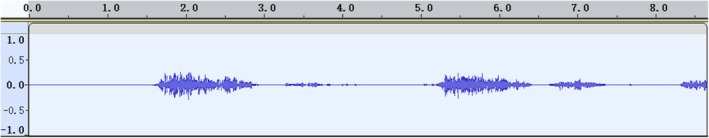
Preprocessed audio recordings of lung sounds (Corresponding Power: 0 dB = 1 pW).

Before standardization, the collected respiratory sounds were difficult to identify due to interference from heart sounds in the three auscultation areas on the left side, which were close to the heart (Figure 3). In contrast, the three auscultation areas on the right side experienced less interference from heart sounds (Figure 4). After undergoing standardization processing, the characteristics of respiratory sounds became evident, resulting in noise reduction and signal amplification effects, leading to clearer waveforms (Figure 5).

### 3.3 Respiratory Sound Cycle Segmentation

**FIGURE 6:**
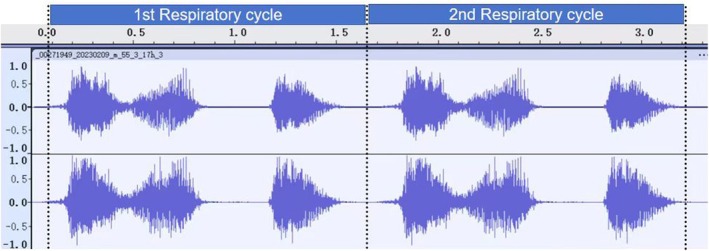
Breath cycle segment (Corresponding Power: 0 dB = 1 pW).

The segmentation of multiple respiratory cycles from an audio file was based on the localization of two adjacent “similar images” in phonocardiography (PCG) (defined as two consecutive images with similar amplitude and duration) (Figure 6).

### 3.4 The Inhalation Phase (S1) and Exhalation Phase (S2) Separation

**FIGURE 7:**
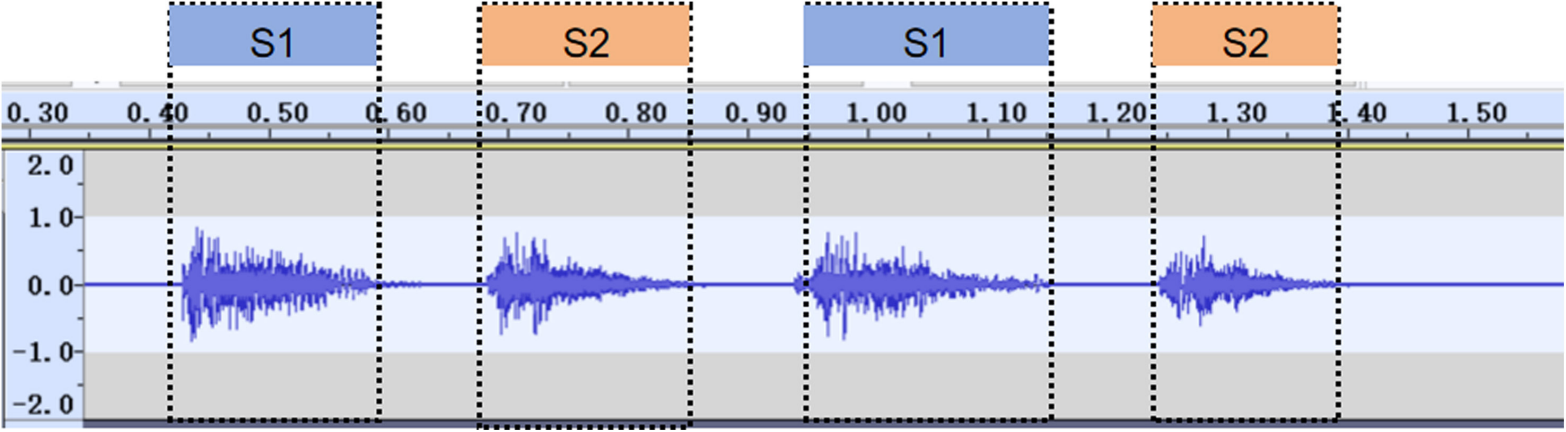
Inhale and exhale separate in every breath cycle (Corresponding Power: 0 dB = 1 pW).

After positioning and segmenting the respiratory cycle, audio data analysis tools can identify the intensity, duration, and frequency‐domain characteristics of S1 and S2 as follows: (I) S1 has a longer duration than S2 and its amplitude was significantly higher than that of S2. (II) Both S1 and S2 have a certain frequency range in physiological terms. (III) In PCG, S2 occurs between adjacent S1 signals. By combining these features, we determined the start and end positions of both S1 and S2 (Figure 7).

### 3.5 Segmentation of Inhalation and Exhalation Phases Based on Label Information

**FIGURE 8:**
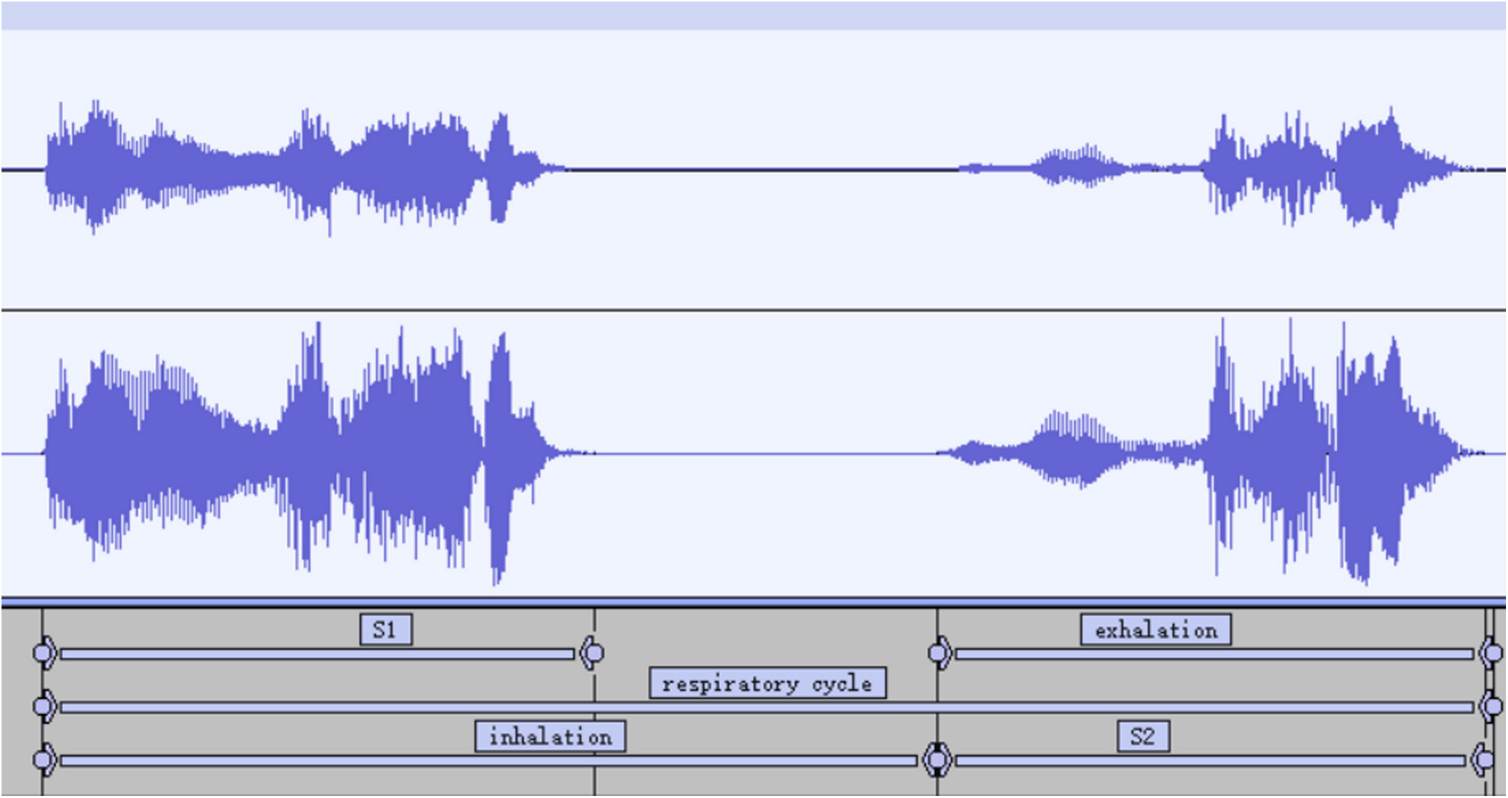
Start and end times of S1 and S2.

The time tag information was extracted from the Respiratory Data Analysis Tool to accurately separate the inhalation and exhalation periods. After identifying and locating S1 and S2, label values were calculated (Figure 8) to precisely clip the recordings. The recorded respiratory sounds include precise start and end times for each breathing cycle.

The amplitude of respiratory sounds can indicate the level of physical activity in the respiratory muscles, and previous studies suggested that this could be valuable for COVID‐19 detection [13]. Sound magnitude was utilized to calculate and filter loudness values, specifically determining the amplitudes of different segments (S1 and S2) within the respiratory cycle. In this study, we computed the average and standard deviation of amplitude features (Maxdb, Mindb, Meandb, and Midledb) for each segment of the respiratory cycle.

### 3.6 Spectrum

Spectrum analysis is the most widely used method in respiratory sound analysis. In this study, Python language was used to perform formula calculations on two segments (S1 and S2).

### 3.7 Cross‐Validation Based on the Respiratory Cycle

In order to validate the accuracy of our segmentation results, a double‐blind validation was also conducted respiratory phase verification with experienced experts in the field of respiratory diseases. This was performed to record the subjects' respiratory rates during the data collection process. See Table 4.

**TABLE 4:** Summary table for extract variables.

	Group	Mean	Std. err.	Std. dev.	95% Conf. interval	
Abosomean	1	0.04	< 0.01	0.02	0.03	0.04
	2	0.04	< 0.01	0.01	0.03	0.052
Peak2valley	1	1.75	0.03	0.27	1.68	1.81
	2	1.98	0.01	0.05	1.95	2.01
Crestfactor	1	10.63	0.61	5.10	9.42	11.84
	2	8.73	0.64	2.14	7.30	10.17
Shapefactor	1	2.70	0.06	0.53	2.57	2.82
	2	2.88	0.07	0.24	2.72	3.04
Marginfactor	1	1535.59	492.70	4151.56	552.93	2518.25
	2	690.62	117.37	389.26	429.11	952.13
Energy	1	2367.00	225.38	1899.13	1917.49	2816.52
	2	4461.66	605.33	2007.65	3112.91	5810.42
f0variation	1	107.34	2.89	24.32	101.58	113.09
	2	84.18	3.33	11.05	76.75	91.61
f0skew	1	0.48	0.06	0.53	0.35	0.60
	2	0.67	0.14	0.45	0.37	0.98
Jitter	1	< 0.01	< 0.01	< 0.01	< 0.01	< 0.01
	2	< 0.01	< 0.01	< 0.01	< 0.01	< 0.01
Duration	1	4.94	0.26	2.18	4.42	5.45
	2	6.64	0.35	1.17	5.85	7.42

## 4 Discussion

Respiratory system diseases are one of the most common health issues affecting people in their daily lives. With a high incidence rate and limited diagnostic methods, there is an urgent need for simple, reliable, and radiation‐free diagnostic techniques. Intelligent auscultation technology for respiratory sounds can play a significant role in predicting respiratory system diseases [14, 15]. However, during the performance of respiratory auscultation in a clinical frontline, even experienced respiratory specialists can be disturbed in the diagnosis of respiratory disorders due to the potential influence of a variety of external factors, which may lead to misdiagnosis or omission.

The artificial intelligence recognition technology can detect acoustic information from respiratory sound signals, encompassing temporal domain features (e.g., energy, average value, and peak value), frequency‐domain features (e.g., spectral energy and frequency peak), and time‐frequency domain features (e.g., wavelet coefficients). Moreover, this method presents the advantages of batch processing and convenience in screening and diagnosing respiratory system diseases [16]. In this study, we developed and validated an automated method for segmenting respiratory sounds and extracted basic acoustic information and sound intensity parameters using our audio data analysis tool. Through data analysis, no significant differences were found in respiratory sound against the respiratory system diseases between those detected by the audio data analysis tool and radiological examinations.

Diagnosis of respiratory diseases can be greatly improved by utilizing intelligent digital auscultation technology. A comprehensive signal analysis method for analyzing challenging recordings of crackles includes five processing modules: (1) motion artifact detection, (2) deep learning denoising network, (3) respiratory cycle segmentation, (4) separation of discontinuous nontonal components from bubble sounds, and (5) crackle peak detection. These methods provide quantifiable analysis of respiratory sounds, enabling clinicians to differentiate various types of crackles based on their timing and severity within the respiratory cycle [17]. To address the occasional misidentification of respiratory sounds by medical professionals, Kim et al. developed an automatic classification system for respiratory sounds by using of deep learning CNN [17]. They successfully categorized 1918 types of respiratory sounds from clinical records into various categories including normal, crackles, wheezes, and rhonchi. By integrating a pre‐trained sequential image feature extractor with the respiratory sound data and CNN classifier, they established a predictive model for accurately classifying respiratory sounds. This innovative model holds great potential in facilitating prompt diagnosis and appropriate treatment of respiratory system diseases [18]. Heitmann et al. used 35.9 h of auscultation audio data from 572 pediatric outpatient patients to develop Deep Breath: a deep learning model for identifying sound features of acute respiratory diseases in children [18]. The model consists of a CNN and a logistic regression classifier, which aggregated estimates recorded from eight chest locations into a single prediction at the patient level. Internal fivefold cross‐validation was performed on the results, and external validation was conducted in three other countries (Senegal, Cameroon, and Morocco). Deep Breath provides an interpretable framework for deep learning to identify objective audio features of respiratory diseases [19]. The major of existing lung sound recognition methods neglect the correlation between temporal and spectral information of lung sounds, resulting in insufficient capture of detailed features. Shi et al. introduced a model that incorporates wavelet feature enhancement and time‐frequency synchronized modeling techniques [17]. This model comprises a dual wavelet analysis module (DWAM), a cubic network, and an attention module. Experimental results on both merged datasets and those from the 2017 International Conference on Biomedical and Health Informatics demonstrate that their proposed framework outperforms existing models by over 1.36% and 4.28%, respectively [20].

Our research shares similarities with theirs, commencing from the rigorous selection of participants based on specific inclusion and exclusion criteria, employing advanced electronic stethoscopes for capturing respiratory sound data, followed by preprocessing of sound signals, extraction of relevant features, and ultimately achieving comprehensive analysis of respiratory sounds. However, it has been suggested in certain studies that harnessing machine learning models for analyzing lung sounds can further explore their diagnostic potential for other diseases related to the respiratory system [18].

The innovation in our respiratory sound signal processing embedded in the utilization of our self‐developed software, Repertory Data Analysis Tool, for data processing. This tool effectively aggregates time labels for respiratory sounds and employs Python language for audio analysis, feature extraction, and the generation of partial spectrograms. In addition, all acoustic parameters can be collected, which also facilitates researchers in diversifying the data.

The value of the integrated system developed in this study lies not merely in the technical integration of modules, but more importantly in its provision of a feasible technical pathway for the clinical application of respiratory sound analysis. By enhancing the automation and user‐friendliness of the entire process, the system is positioned to lower the barrier to implementation of respiratory sound analysis technology, thereby promoting its adoption in primary care settings and remote monitoring scenarios. This advancement will ultimately enable broader utility in early screening, diagnosis, and long‐term monitoring of respiratory diseases.

This study has notable limitations constraining its generalizability. Data from 84 patients at one site under controlled conditions limits broader applicability. Unaddressed gaps include interdevice consistency, ambient noise impacts in real‐world settings, and performance across diverse body types or disease states—critical for clinical/telemonitoring use. Future work should address these to enhance translational value.

## 5 Conclusion

The integration of electronic stethoscope and artificial intelligence technology has facilitated the digital auscultation technique, enabling the digitized collection of respiratory sounds and precise identification of inspiratory phase (S1) and expiratory phase (S2). The analysis results obtained through artificial intelligence technology on respiratory sounds demonstrate robust scientific stability. This approach provides objective evidence for respiratory auscultation and noise assessment, thus holding significant importance in clinical research.

### 5.1 Limitations

Despite our efforts to minimize noise during data collection, significant amounts of noise were present due to uncontrollable factors such as heart sounds, poor stethoscope fit on thin patients, and external environmental factors. Additionally, manually cutting audio files at their beginnings and endings during sound processing could potentially affect data analysis results.

## Author Contributions

**F.J., N.H.R., and C.X.L.** data collection management, formal analysis, resources, ethical application, and writing original draft. **D.Y.L.:** formal analysis, validation, project management, resources, and writing original draft. **W.W.M.:** data collection and data classification management. **X.F. and S.Y.:** methodology, grant acquisition, project management, and review and editing.

## Ethics Statement

The ethics committee at the Ethics Committee of Shanghai Changhai Hospital approved the investigation (CHEC2024‐049). The written informed consent has been obtained from all participants.

## Consent

The authors have nothing to report.

## Conflicts of Interest

The authors declare no conflicts of interest.

## Data Availability

The authors will provide the original data supporting the conclusions of this article without any unnecessary withholding.
